# Survival and Hemodynamic Effects of Parenteral Prostanoids in High‐Altitude Group 1 Pulmonary Hypertension: A Retrospective Cohort Study

**DOI:** 10.1155/pm/2867844

**Published:** 2026-05-19

**Authors:** Rafael Conde-Camacho, Eduardo Tuta-Quintero, Mauricio González, Wendy Rubiano, Emily Rincón, Katherine Diaz, Luis F. Giraldo-Cadavid

**Affiliations:** ^1^ Biosciences Doctoral, Universidad de La Sabana, Chía, Colombia, unisabana.edu.co; ^2^ Department of Pulmonary Hypertension, Fundación Neumológica Colombiana, Bogotá, Colombia; ^3^ School of Medicine, Universidad de La Sabana, Chía, Colombia, unisabana.edu.co; ^4^ Medical Department, Fundación Neumológica Colombiana, Bogotá, Colombia

**Keywords:** parenteral prostanoids, pulmonary hypertension, risk factor, survival

## Abstract

**Background:**

In Latin America, the evidence regarding the use of prostanoids at high altitude in patients with Group 1 pulmonary hypertension (PH‐1) is limited. Therefore, it is essential to describe the clinical characteristics and outcomes associated with prostanoid treatment in this population.

**Methods:**

This retrospective study involved patients with PH‐1. Hemodynamic and clinical variables were compared before the initiation of prostanoids and during clinical follow‐up. Overall survival was analyzed using Kaplan–Meier estimation, with survival rates calculated at 3 and 5 years. The strength of the association for each variable concerning the proposed outcomes was estimated by calculating the odds ratio (OR).

**Results:**

The study included 55 patients (mean age 34.6 ± 10.3 years), 83.6% women, all receiving triple combination therapy with endothelin receptor antagonists (ERAs), phosphodiesterase Type 5 inhibitors, and parenteral prostacyclin analogues. Idiopathic pulmonary arterial hypertension was present in 62%, and 60% were classified as intermediate risk at baseline. Median time from PH‐1 diagnosis to prostacyclin initiation was 8.96 months (IQR 1.88–41.42). At 1 year, complete follow‐up data were available for 49 patients. St. George′s Respiratory Questionnaire scores improved significantly from 49.0 to 36.5 at 1 year and 31.5 at the last follow‐up (*p* < 0.001). Cardiac index increased from 2.24 to 2.96 L/min/m^2^ at 1 year (*p* < 0.001), whereas mixed venous oxygen saturation improved significantly. In survivors, the 6‐min walk test distance increased by 53.5 m, and pulmonary vascular resistance decreased by 3.8 WU. Diffusing capacity for carbon monoxide (DLCO) < 25% was associated with an OR of 7.35 (95% CI: 1.28–41.92; *p* = 0.015), and tricuspid annular plane systolic excursion to pulmonary artery systolic pressure ratio (TAPSE/PASP) < 0.10 mm/mmHg had an OR of 5.78 (95% CI: 0.64–52.03; *p* = 0.088). Female sex had an OR of 0.13 (95% CI: 0.02–0.78; *p* = 0.016). Three‐ and five‐year survival rates were 87.2% and 78.1%, respectively.

**Conclusion:**

Parenteral prostanoids in patients with PH‐1 demonstrate improvements in hemodynamic variables during clinical follow‐up. A DLCO < 25% and a TAPSE/PASP < 0.10 mm/mmHg were associated with an increased risk of mortality, whereas female sex was identified as a protective factor.

## 1. Introduction

Pulmonary hypertension (PH) is defined as a mean pulmonary arterial pressure (mPAP) > 20 mmHg in the pulmonary circulation, resulting from endothelial dysfunction, inflammation, and pulmonary vascular remodeling, or from elevated precapillary pressures relative to postcapillary pressures [1–3]. PH predominantly affects women aged 30–60 years, with an estimated prevalence of 15–50 cases per million in the United States and Europe [3, 4]. Group 1 pulmonary hypertension (PH‐1) is characterized by abnormal proliferation of smooth muscle cells and endothelial dysfunction, producing distinct pathophysiological and hemodynamic features in the absence of other causes of precapillary PH [5–7].

Treatment of PH‐1 is guided by disease severity at diagnosis and during follow‐up, patient tolerance, and potential adverse effects, with risk stratification into low, intermediate, or high categories based on mortality predictors [4–7]. Right ventricular function is assessed using brain natriuretic peptide (BNP) or N‐terminal pro‐BNP, as well as echocardiography or right heart catheterization [1, 2]. Pharmacological management includes endothelin receptor antagonists, phosphodiesterase Type 5 inhibitors, guanylate cyclase stimulators, and prostacyclin receptor agonists or analogues, aiming to improve functional status, hemodynamics, and quality of life [6–9].

Prostanoids are the treatment of choice in advanced disease (New York Heart Association [NYHA] functional Classes III–IV), producing vasodilation, antiproliferative effects on the extracellular matrix, anticoagulation, and anti‐inflammatory activity, with beneficial effects on the pulmonary vasculature and right ventricular function [7, 10–13]. In Latin America, access to parenteral prostanoids for the treatment of PH‐1 is limited due to high cost and heterogeneous healthcare systems, and published clinical experience is scarce [12, 13]. To address this gap, the present study is aimed at describing the clinical characteristics, functional and hemodynamic outcomes, and survival of PH‐1 patients receiving combination therapy including parenteral prostanoids, providing real‐world evidence in a population living at high altitude.

## 2. Methods

A retrospective study was conducted on patients with a confirmed diagnosis of PH‐1 receiving treatment with Type I endothelin antagonists and phosphodiesterase Type 5 inhibitors who experienced disease progression requiring the administration of parenteral prostanoids (triple therapy). Patients were consecutively evaluated upon entering the vascular disease program, with follow‐up assessments conducted every 12 months after initiating treatment with epoprostenol or treprostinil, from January 2013 to December 2023. This study took place at a referral center for vascular diseases at the Fundación Neumológica Colombiana, located in Bogotá, Colombia (2640 m above sea level). Although some patients were referred from other regions of the country, all clinical follow‐up and treatment assessments were performed at this high‐altitude center.

### 2.1. Eligibility Criteria

Patients aged over 16 years with a hemodynamic diagnosis of PH‐1 associated with idiopathic, hereditary, drug‐induced toxins, connective tissue diseases, acquired immunodeficiency virus (HIV), congenital heart disease (atrial septal defect, ventricular septal defect, and patent ductus arteriosus), or portopulmonary hypertension were included. Patients with concomitant conditions that limited periodic follow‐up, those with Group 2 and 3 PH, renal failure requiring dialysis, hepatic insufficiency (Child–Pugh B or C), or active cancer were excluded.

### 2.2. Variables

The diagnosis of PH‐1 was defined by a mPAP > 20 mmHg, pulmonary vascular resistance (PVR) > 3 Wood units (WU), and Pulmonary capillary wedge pressure (PCWP) ≤ 15 mmHg, according to the European Society of Cardiology/European Respiratory Society (ESC/ERS) [14]. All patients had a hemodynamically confirmed diagnosis of PH‐1 before initiation of parenteral prostacyclin analogue therapy and were escalated from dual oral therapy to triple therapy because of clinical progression. Variables considered included age, sex, type of endothelin receptor antagonist, phosphodiesterase Type 5 inhibitor, type and dose of prostanoid, and classification of PH‐1 according to etiology. Risk was assessed based on the simplified version of the ESC/ERS guidelines; hemodynamic variables include: mPAP, PVR, PCWP, right atrial area, cardiac index (CI), mixed venous oxygen saturation (SvO2), and the tricuspid annular plane systolic excursion/pulmonary artery systolic pressure (TAPSE/PASP) ratio. Additionally, functional class was described according to the NYHA, diffusing capacity for carbon monoxide (DLCO), BNP, and the St. George′s Respiratory Questionnaire (SGRQ). The 6‐min walk test (6MWT) was conducted on a flat, leveled, 30‐m–long course located within the healthcare institution, free of obstacles and pedestrian movement.

### 2.3. Sample Size

All subjects meeting the selection criteria were included, and data were collected by trained personnel reviewing clinical records, right heart catheterization, and reports from acute events. Data review was performed by two researchers to minimize potential transcription errors.

### 2.4. Statistical Analysis

Qualitative variables were described using absolute and relative frequencies, whereas quantitative variables were presented according to their distribution characteristics as means with standard deviations for normally distributed data or as medians with interquartile ranges for nonnormally distributed data [15]. Bivariate analysis was conducted to compare clinical outcomes before the initiation of parenteral prostanoids and at 1 year of follow‐up. Additionally, functional and hemodynamic parameters were assessed at study baseline and throughout the 5‐year clinical follow‐up, with results analyzed separately for patients who survived and those who died. For categorical quantitative variables, the McNemar test was used; for continuous variables, either the Student′s *t*‐test or the Wilcoxon signed‐rank test was applied, depending on the distribution and nature of the variable [15]. Overall survival was analyzed using Kaplan–Meier estimation, calculating survival rates at 3 and 5 years. Survival was evaluated from the date of prostanoid initiation to the event of interest (death) or the end of the follow‐up period [15].

The strength of association of each variable regarding the proposed outcomes was estimated by calculating the odds ratio (OR) [15]. A *p* value of < 0.005 was considered statistically significant. Statistical analyses were conducted using SPSS Version 25 and Stata 13, licensed to a researcher.

### 2.5. IRB Approval

The study was conducted in accordance with the principles of the current Helsinki Declaration, as well as local, regional, and international regulations pertaining to clinical research, including Colombian Law on Biomedical Research. Ethical approval was obtained from the Medical Ethics Committee of the Fundación Neumológica Colombiana (Approval Number: 202010‐25808). Prior to participating in the study, all participants provided written informed consent, and the confidentiality of their data was strictly maintained throughout the study.

## 3. Results

The study included 55 patients with an average age of 34.6 years (SD: 10.3), of whom 83.6% (46/55) were women (Table 1). All patients initiated a combination therapy with endothelin receptor antagonists, phosphodiesterase Type 5 inhibitors, and parenteral prostanoids. Among endothelin receptor antagonists, 55% (30/55) received bosentan, 33% (18/55) ambrisentan, and 13% (7/55) macitentan. Regarding phosphodiesterase Type 5 inhibitors, 91% (50/55) received sildenafil and 9% (5/55) tadalafil. Among parenteral prostanoids, 73% (40/55) were treated with treprostinil and 27% (15/55) with epoprostenol. Complete clinical follow‐up data at 1 year were available for 49 patients. The reduction reflects losses during follow‐up, primarily due to early mortality and, to a lesser extent, geographic barriers to follow‐up. Sixty‐two percentage (34/55) of the patients were classified with idiopathic PH. Seventeen patients had congenital heart disease–associated pulmonary arterial hypertension, including 24% (4/17) with systemic‐to‐pulmonary shunts and persistent PH, 47% (8/17) with small or coincidental defects, and 29% (5/17) with corrected congenital heart defects. At baseline, 60% (33/55) of patients were classified as intermediate risk and 40% (22/55) as high risk. The median time from PH‐1 diagnosis to initiation of parenteral prostacyclin analogue therapy was 8.96 months (IQR 1.88–41.42).

**Table 1:** General characteristics of the population.

Age years, mean (SD)	34.6 (10.3)
Female, *n* (%)	46 (84)
Weight kg, mean (SD)	63.2 (13.1)
Height cm, mean (SD)	158.6 (6.9)
BMI kg/m^2^, mean (SD)	25.1 (4.3)
Comorbidities, *n* (%)	
Asthma, *n* (%)	5 (9)
Cardiovascular disease, *n* (%)	3 (5)
Arterial hypertension, *n* (%)	4 (7)
Obstructive sleep apnea, *n* (%)	3 (5)
Subclassification of pulmonary hypertension, *n* (%)	
Idiopathic	34 (62)
Connective tissue disease	2 (4)
HIV infection	1 (2)
Portal hypertension	1 (2)
Congenital heart disease, *n* (%)∗	
Systemic–to–pulmonary shunt	4 (24)
Small/coincidental defects	8 (47)
Birth defect closure	5 (29)
Stratification risk, *n* (%)	
Intermediate	33 (60)
High	22 (40)
FVC L, mean (SD)	3.2 (0.8)
FEV_1_ L, mean (SD)	2.6 (0.8)
DLCO %, mean (SD)	24.5 (6.1)
PH, mean (SD)	7.43 (0.7)
PaCO_2_ mmHg, mean (SD)	29.4 (2,8)
HCO_3_‐, mean (SD)	19.4 (1.7)
PaO_2_ mmHg, mean (SD)	59.1 (9.1)
SaO_2_ %, mean (SD)	89.4 (6.1)
FiO_2_ %, mean (SD)	28.0 (2.0)
Hemoglobin g/dL, mean (SD)	15.3 (4.3)
LVEF %, mean (SD)	56 (9.3)
TAPSE mm, mean (SD)	10.3 (7.9)
Right atrial area (mL/m²) mmHg, mean (SD)	36.5 (17.9)
PASP mmHg, mean (SD)	93.7 (29.4)
TAPSE/PASP ratio, mean (SD)	0.10 (0.1)

*Note:* Asterisk “∗” denotes 17 patients with congenital heart disease.

Abbreviations: BMI, body mass index; DLCO, diffusing capacity of the lung for carbon monoxide; FEV_1_, forced expiratory volume in one second; FiO_2_, fraction of inspired oxygen; FVC, forced vital capacity; HCO_3_, bicarbonate concentration in blood; LVEF, left ventricular ejection fraction; m, mean; PaCO_2_, partial pressure of carbon dioxide in arterial blood; PaO_2_, partial pressure of oxygen in arterial blood; PH, pulmonary hypertension; PASP, pulmonary artery systolic pressure; SaO_2_, arterial oxygen saturation; SD, standard deviation; TAPSE, tricuspid annular plane systolic excursion.

The score on the St. George′s questionnaire decreased from 49.04 points (SD: 19.6) prior to the initiation of prostanoids to 36.51 (SD: 12.3) at 1 year of follow‐up and 31.53 (SD: 13.4) at the last follow‐up (*p* < 0.001) (Table 2). The PVR decreased from 16.74 WU (SD: 8.3) prior to prostanoid initiation to 12.59 (SD: 6.7) at 1 year of follow‐up and 14.34 WU (SD: 4.4) at the last follow‐up (*p* = 0.065). Pulmonary capillary pressure increased from 11.31 mmHg (SD: 3.0) to 13.0 mmHg (SD: 2.6) (*p* = 0.004), whereas SvO2 improved from 62.08% (SD: 7.2) to 67.6% (SD: 5.1) (*p* = 0.032). mPAP showed numerical reductions during follow‐up, although these changes did not reach statistical significance (67.62 vs. 60.72 mmHg, *p* = 0.275). The CI improved from 2.24 L/min/m^2^ (SD: 0.5) prior to prostanoid initiation to 2.96 (SD: 1.1) at 1 year of follow‐up; however, it decreased to 2.33 L/min/m^2^ at the last follow‐up (*p* < 0.001).

**Table 2:** Functional and hemodynamic variables in clinical follow‐up.

	Prior to prostanoid *n* = 55	Follow‐up year *n* = 49	*p*
6MWT m, mean (SD)	446.67 (115.8)	483.49 (101.5)	0.119
SGRQ, mean (SD)	49.04 (19.6)	36.51 (12.3)	0.000
Pulmonary artery systolic pressure mmHg mean (SD)	102.44 (28.9)	93.13 (20.9)	0.213
Pulmonary artery diastolic pressure mmHg mean (SD)	46.69 (16.8)	40.53 (12)	0.388
mPAP mmHg, mean (SD)	67.62 (19.1)	60.49 (14.6)	0.275
Right ventricular pressure mmHg cm^2^, mean (SD)	11.8 (4.8)	11.91 (4.4)	0.967
PCWP mmHg, mean (SD)	11.31 (3)	12.81 (3.1)	0.004
PVR, Wood units mean (SD)	16.74 (8.3)	12.59 (6.7)	0.065
CI TD L/min/m^2^, mean (SD)	2.24 (0.5)	2.96 (1.1)	0.000
CO L/min, mean (SD)	3.89 (1.1)	4.5 (1.2)	0.097
SvO_2_ %, mean (SD)	62.08 (7.2)	64.57 (7.2)	0.032
BNP pg/mL, mean (SD)	274.5 (332.8)	246.4 (294)	0.172

Abbreviations: 6MWT, 6‐minute walk test; BNP, B‐type natriuretic peptide; CI, cardiac index; cm^2^, square centimeters; CO, cardiac output; mmHg, millimeters of mercury; mPAP, mean pulmonary arterial pressure; PCP, pulmonary capillary pressure; pg/mL, picograms per milliliter; PCWP, Pulmonary capillary wedge pressure; PVR, pulmonary vascular resistance; SvO_2_, mixed venous oxygen saturation; SD, standard deviation; SGRQ, St. George′s Respiratory Questionnaire; SP, systolic pressure; WU, Wood units.

**Figure 1:**
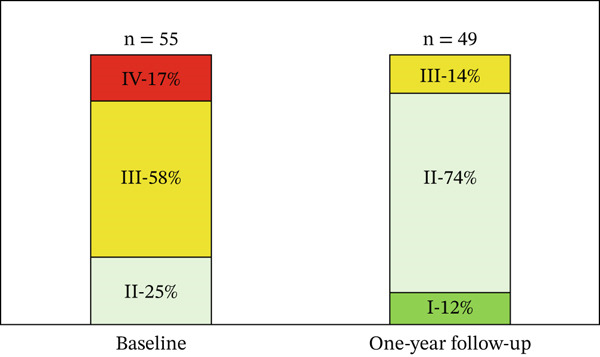
Functional class measured by the New York Heart Association.

Functional class measured by NYHA is described in Figure 1. In patients who survived, the distance walked in the 6MWT increased by 53.5 meters (447.6 vs. 501.1; *p* < 0.001), the St. George′s questionnaire score decreased by 13 points (47.62 vs. 34.6; *p* = 0.008), mPAP decreased by 8.4 mmHg (67.3 vs. 58.9; *p* < 0.001), PVR decreased by 3.8 WU (15.5 vs. 11.7; *p* = 0.001), and CI increased by 0.8 L/min/m^2^ (2.3 vs. 3.1; *p* = 0.002) (Table 3). Patients who died showed smaller and nonsignificant changes in these variables, with mPAP decreasing from 68.54 to 65.75 mmHg (*p* = 0.088), PVR from 20.42 to 15.68 WU (*p* = 0.053), and CI increasing from 2.02 to 2.76 L/min/m^2^ (*p* = 0.463).

**Table 3:** Functional and hemodynamic characteristics in clinical follow‐up among living and dead patients.

	Alive *n* = 45			Dead *n* = 10		
	Baseline	Follow‐up 5 years	*p*	Baseline	Follow‐up 5 years	*p*
6MWT m, mean (SD)	447.6 (123.2)	501.11 (98.2)	< 0.001	443.69 (92)	424 (95.1)	0.148
SGRQ, mean (SD)	47.62 (18.6)	34.59 (12.6)	0.008	53.62 (22.8)	43 (9)	0.161
Pulmonary artery systolic pressure (mmHg) mean (SD)	101.17 (27.8)	91.04 (21.6)	0.011	106.46 (33)	100.19 (17.6)	0.815
Pulmonary artery diastolic pressure (mmHg) mean (SD)	46.51 (16.8)	38.8 (11.3)	< 0.001	47.23 (17.3)	46.38 (13.4)	0.110
mPAP mmHg, mean (SD)	67.33 (19.1)	58.93 (14.5)	< 0.001	68.54 (20)	65.75 (14.7)	0.088
Right ventricular pressure (mmHg) cm^2^, mean (SD)	10.83 (4.2)	11.16 (4)	0.876	14.77 (5.4)	14.44 (4.8)	0.373
PCWP mmHg, mean (SD)	10.74 (2.6)	12.61 (3.4)	0.322	13.36 (3.4)	13.45 (1.8)	0.656
PVR, Wood units mean (SD)	15.54 (6.7)	11.67 (4.2)	0.001	20.42 (11.5)	15.68 (11.7)	0.053
CI TD L/min/m^2^, mean (SD)	2.31 (0.6)	3.02 (1.2)	0.002	2.02 (0.2)	2.76 (0.3)	0.463
CO L/min, mean (SD)	4.01 (1.1)	4.52 (1.3)	0.889	3.41 (0.9)	4.45 (0.1)	0.764
SvO_2_ %, mean (SD)	62.95 (7.3)	65.74 (6.3)	0.059	59.09 (6)	60.63 (9.1)	0.289
BNP pg/mL, mean (SD)	226.22 (211.5)	166.74 (150.3)	0.601	415.34 (544)	515.25 (478.5)	0.048

Abbreviations: 6MWT, 6‐minute walk test; BNP, B‐type natriuretic peptide; CI, cardiac index; cm^2^, square centimeters; CO, cardiac output; PCWP, Pulmonary capillary wedge pressure; mmHg, millimeters of mercury; mPAP, mean pulmonary arterial pressure; PCP, pulmonary capillary pressure; pg/mL, picograms per milliliter; PVR, pulmonary vascular resistance; SvO_2_, mixed venous oxygen saturation; SD, standard deviation; SGRQ, St. George′s Respiratory Questionnaire; SP, systolic pressure; WU, Wood units.

**Figure 2:**
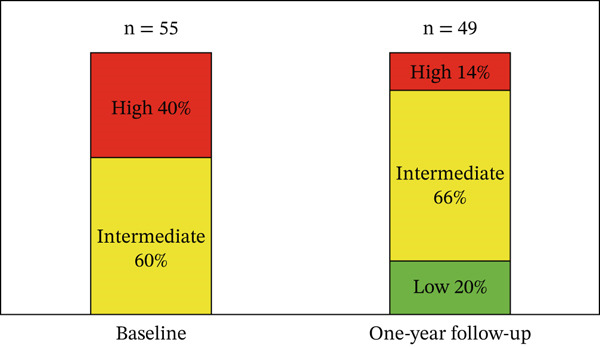
Risk stratification according to the simplified version of the ESC/ERS guidelines.

**Figure 3:**
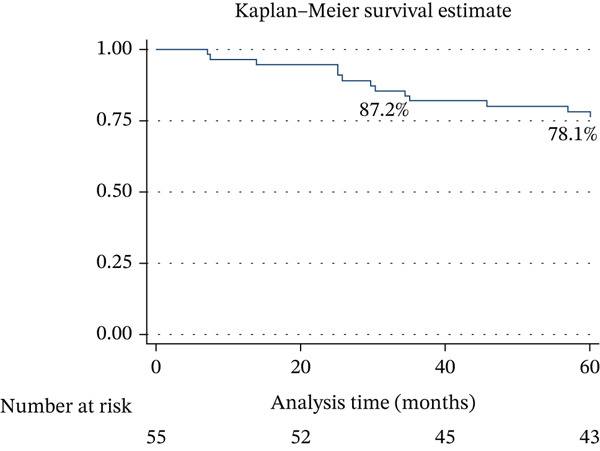
Survival at 3 and 5 years.

High risk decreased by 26%, whereas low risk increased by 20% at 1 year of follow‐up (Figure 2). The survival rate at 3 years of follow‐up was 87.2%, and at 5 years, it was 78.1% (Figure 3). DLCO < 25% had an OR of 7.35 (95% CI: 1.28–41.92; *p* = 0.015), and TAPSE/PASP < 0.10 mm/mmHg had an OR of 5.78 (95% CI: 0.64–52.03; *p* = 0.088). Female sex had an OR of 0.13 (95% CI: 0.02–0.78; *p* = 0.016) (Table 4).

**Table 4:** Risk factors associated with mortality.

	OR	95% CI	*p* value
DLCO <25%	7.35	1.28–41.92	0.015
TAPSE/PASP <0.10 mm/mmHg	5.78	0.64–52.03	0.088
mPAP >40 mmHg	1.81	0.37–1.92	0.640
Age >34 years	1.03	0.23–4.58	0.970
PaO_2_ >60 mmHg	0.98	0.16–5.82	0.982
Cardiac index >2 L/min/m^2^	0.48	0.09–2.49	0.377
Female sex	0.13	0.02–0.78	0.016

Abbreviations: DLCO, diffusing capacity of the lung for carbon monoxide; mPAP, mean pulmonary arterial pressure; PaO_2_, partial pressure of oxygen in arterial blood; TAPSE/PASP, tricuspid annular plane systolic excursion/pulmonary artery systolic pressure.

## 4. Discussion

This study evaluated the impact of parenteral prostanoid therapy on survival, functional tests, and hemodynamic variables in patients with PH‐1. Our results show a survival rate of 87.2% at 3 years and 78.1% at 5 years, with significant improvements in PVR, CI, mPAP, and SvO2 during clinical follow‐up, particularly among patients who survived. DLCO < 25% and TAPSE/PASP < 0.10 mmHg were associated with an increased risk of mortality, whereas female sex was a protective factor [10, 16–20].

After initiating prostanoids, an increase in the number of patients classified as low risk was observed, along with improvements in functional class according to NYHA and an increase in 6MWD among survivors. These findings are consistent with previous studies that documented hemodynamic improvements and significant changes in risk classification [10, 16–20].

Badagliacca et al. [18] evaluated long‐term survival in 267 consecutive patients with idiopathic PH‐1, reporting survival rates of 90%, 79%, 70%, 55%, and 42% at 1, 3, 5, 10, and 15 years, respectively. They identified mPAP ≤ 35 mmHg, the time from diagnosis to prostanoid initiation, and the maximum dose as independent predictors of survival [18]. Our results demonstrate comparable survival and suggest that reductions in mPAP are associated with a better response to triple therapy including parenteral prostanoids.

At a population level, approximately 140 million people live at high altitudes (> 2500 m), and data on PH in this context are limited [21, 22]. Our patients showed elevated mPAP and minimal changes during follow‐up, likely related to physiological adaptations to hypoxia. Fakhri et al. [23] reported that patients with PH‐1 living at high altitude have higher mPAP, higher PVR, and greater 6MWD, effects independent of age, sex, body mass index, oxygen use, race, or other factors [23–25]. Chronic hypoxic exposure also induces persistent vascular remodeling in small pulmonary arteries and veins [24–27], contributing to sustained PH. Aldashev et al. [26] reported a prevalence of PH at high altitude ranging from 5% to 18%.

In our cohort, 30% of patients had congenital heart disease, a higher proportion than reported in other series (5%–16%) [28], and these patients presented elevated mPAP. Evidence suggests that early initiation of prostanoids allows achieving therapeutic targets of mPAP < 25 mmHg, whereas delays in treatment are associated with unfavorable outcomes [11, 18, 29].

Bartolome et al. [17] reported survival rates of 84%, 77%, and 66% at 1, 2, and 3 years, respectively, and de Lang et al. [16] reported 89%, 71%, and 66% at the same intervals. The differences reflect variations in population and dosing regimens. Our data highlight the importance of predicting worse outcomes in high‐risk or urgent patients compared with those in elective or intermediate conditions, emphasizing timely referral to specialized PH‐1 centers.

Synthetic prostanoids that stimulate the IP receptor induce vasodilation, inhibit platelet aggregation, and exert anti‐inflammatory effects [30, 31]. Epoprostenol improves survival, 6MWD, and functional class in PH‐1 [30–32], but requires continuous infusion. Treprostinil, with a longer half‐life and good tolerability, was used in > 50% of our cohort, showing outcomes comparable with epoprostenol [33–35].

The mean age of our patients was 34.6 years, lower than in other series (> 40 years), which explains why 19% were in functional Classes I–II, and the average 6MWD was 424 meters. Previous studies indicate that younger patients, despite a worse baseline hemodynamic profile, have better functional class, NT‐proBNP levels, and longer walking distances [36]. In our deceased patients, hemodynamic improvements were smaller and not significant, highlighting the need for early intervention and close follow‐up.

### 4.1. Limitations

The results of this observational single‐center study have several limitations. First, the relatively small sample size and limited number of mortality events reduced the statistical power for multivariable survival analyses, preventing the use of a Cox regression model adjusted for potential confounders; therefore, the mortality‐associated factors presented should be interpreted as exploratory findings derived from bivariate analysis. Second, losses during follow‐up, partly attributable to early mortality and to patients lost to follow‐up due to geographic barriers and difficulties in maintaining regular evaluations at our high‐altitude referral center, may have influenced longitudinal comparisons and outcome interpretation, while detailed longitudinal prostanoid dose adjustments were not consistently available in the database. However, data collection was performed by trained members of the research group, and a double verification process was applied during transcription to the electronic database to minimize transcription bias.

The altitude at which the study was conducted (2640 m above sea level) may represent a limitation, since pathophysiological variables at altitudes above 2500 m can differ from those observed in patients residing at sea level. Prospective multicenter studies are needed to confirm the effectiveness and safety of prostanoids in patients with PH‐1 living at high altitude.

## 5. Conclusion

Parenteral prostanoids in patients with PH‐1 were associated with improvements in hemodynamic variables such as mPAP, PVR, PCWP, right atrial area, and CI during clinical follow‐up, particularly among survivors compared with patients who died. A DLCO < 25% and TAPSE/PASP < 0.10 mm/mmHg were associated with an increased risk of mortality, whereas female sex appeared to be a protective factor associated with lower mortality risk. Although these findings should be interpreted cautiously due to the limited sample size and single‐center design, they highlight the potential prognostic value of hemodynamic and functional parameters in identifying high‐risk patients.

## Author Contributions

Conceptualization: R.C‐C., E.T‐Q., and M.G.; methodology: R.C‐C., W.R., E.R., and E.T‐Q.; software: K.D.; validation: R.C‐C. and M.G.; formal analysis: R.C‐C.; investigation: L.F.G‐C., W.R., and E.R.; resources: E.T‐Q. and L.F.G‐C.; data curation: R.C‐C. and L.F.G‐C.; writing—original draft preparation: R.C‐C., E.T‐Q., K.D., M.G., L.F.G‐C., W.R., and E.R.; writing—review and editing: R.C‐C. and K.D.; visualization: R.C‐C. and E.T‐Q.; supervision: M.G.

## Funding

This study was supported by Universidad de La Sabana (10.13039/501100010628; MED‐326‐2022).

## Conflicts of Interest

The authors declare no conflicts of interest.

## Data Availability

The data that support the findings of this study are available from the corresponding author, Rafael Conde‐Camacho, upon reasonable request.
